# Vitamin D suppresses cellular pathways of diabetes complication in liver

**DOI:** 10.22038/ijbms.2019.36054.8584

**Published:** 2019-06

**Authors:** Hoda Derakhshanian, Mahmoud Djalali, Mohammad Hassan Mohammad Hassan, Ehsan Alvandi, Mohammad Reza Eshraghian, Abbas Mirshafiey, Hoda Nadimi, Samane Jahanabadi, Mahnaz Zarei, Abolghassem Djazayery

**Affiliations:** 1 Dietary Supplements and Probiotic Research Center, Alborz University of Medical Sciences, Karaj, Iran; 2 Department of Biochemistry, Genetics and Nutrition, School of Medicine, Alborz University of Medical Sciences, Karaj, Iran; 3 Department of Cellular and Molecular Nutrition, School of Nutritional Sciences and Dietetics, Tehran University of Medical Sciences, Tehran, Iran; 4 Department of Biostatistics, School of Public Health, Tehran University of Medical Sciences, Tehran, Iran; 5 Department of Pathobiology, School of Public Health, Tehran University of Medical Sciences, Tehran, Iran; 6 Department of Pharmacy, Shahid Sadoughi University of Medical Sciences, Yazd, Iran; 7 Department of Community Nutrition, School of Nutritional Sciences and Dietetics, Tehran University of Medical Sciences, Tehran, Iran

**Keywords:** Advanced glycation end – products, Cholecalciferol, Diabetes complications, Hexosamine pathway Vitamin D

## Abstract

**Objective(s):**

The aim of this study was to investigate the effect of vitamin D on glucose metabolism, as well as the expression of five key genes involved in the development of diabetes complications in liver tissue of diabetic rats.

**Materials and Methods:**

Twenty-four male Sprague–Dawley rats were randomly divided into three groups (8 rats in each group). The first group served as control and the other two groups received an intraperitoneal injection of 45 mg/kg streptozotocin to develop diabetes. Groups were treated for four weeks either with placebo or vitamin D (two injections of 20000 IU/kg). Thereafter, serum levels of glucose, insulin and HbA1c were assessed. Liver tissue was examined for the level of advanced glycation end products (AGEs) and the gene expression of AGE cellular receptor (AGER), glyoxalase-1 (GLO-1), aldose reductase (AR), O-linked N-acetylglucosamine transferase (OGT) and glutamine/ fructose-6-phosphate aminotransferase (GFAT).

**Results:**

Vitamin D injection resulted in a significant increase in plasma level of 25-hydroxycholecalciferol, which could improve hyperglycemia about 11% compared to placebo-receiving diabetic rats (*P*=0.005). Insulin level increased as a result of vitamin D treatment compared to control (3.31±0.65 vs. 2.15±0.79; *P*= 0.01). Serum HbA1c and liver AGE concentrations had a slight but insignificant reduction following vitamin D intake. Moreover, a significant decline was observed in gene expression of AGER and OGT in liver tissue (*P*=0.04 and *P*<0.001 respectively).

**Conclusion:**

Vitamin D might contribute in ameliorating diabetes complications not only by improving blood glucose and insulin levels, but also by suppressing AGER and OGT gene expression in the liver.

## Introduction

Global prevalence of diabetes mellitus has been reported in more than 415 million adult populations in 2015, and it is estimated that this figure, by more than 50% growth, will reach 642 million people by 2040. For this reason, the International Diabetes Federation (IDF) has symbolically named Diabetes as the third most populous country in the world. Despite different methods and medications to control blood glucose, diabetes complications such as heart disease, kidney and nerve damages impose enormous medical expenses on the governments, such that most countries spend 5 to 20% of their total health expenditure on diabetes control (1, 2). To date, several mechanisms have been proposed for the etiology and metabolic pathways involved in diabetic complications. The most important pathways include: 1) the pathway for production of advanced glycation end products (AGE) in cells, 2) Polyols pathway, 3) Hexosamine biosynthesis pathway (HBP), 4) the protein kinase C pathway, and 5) Reactive oxygen species (ROS) mitochondrial pathway (3, 4). Several strategies have been explored to decrease the complications of diabetes. Although these strategies do not result in the treatment of the disease, they can considerably decrease health costs and increase life expectancy and quality of life of diabetic patients. Modification of lifestyle and especially an appropriate diet is one of the primary solutions in this regard (5). Receiving accurate and balanced macronutrients and micronutrients play a significant role in diabetes control -as a chronic illness associated with metabolic disorders (6, 7). Vitamin D is one of the micronutrients whose relationship with diabetes has been taken into consideration recently. Several studies have shown that the incidence of type I and type II diabetes is associated with vitamin D deficiency (8, 9). Some studies have shown that receiving vitamin D supplement is associated with reduced complications such as diabetic nephropathy and retinopathy (10-12). 

Considering the high prevalence of both diabetes and vitamin D deficiency in the world, the scrutiny of their relationship is of paramount importance. But, the existing evidences are controversial and inconclusive. For this purpose, the present study focused on the cellular role of vitamin D and its precise mechanism of action in reducing the complications of diabetes. Therefore, some of the most important components of pathways involved in diabetic complications were selected for further study. These components included: 1) AGE receptor (AGER) that is activated through binding to glycated compounds that are produced through non-enzymatic reactions following a high concentration of sugar in the blood, and leads to increased production of nuclear factor-kappa B (NF-κB) and its subsequent inflammatory complications. 2) Glyoxalase-1 (GLO-1) is an enzyme that decreases the AGE concentration and the possibility of their attachment to AGER by metabolizing AGE and hence decreases the activation of inflammatory pathway. 3) Aldose reductase (AR) that is the main enzyme of polyols pathway and is responsible for the production of compounds like sorbitol and galactitol and complications caused by them in diabetes. 4) O-linked N-acetylglucosamine transferase (OGT) that is an enzyme that attaches glucosamine produced in the hexosamine pathway to protein compounds and by increasing the production of glycosylated proteins contributes in the development of diabetic complications. 5) Glutamine: fructose-6-phosphate aminotransferase (GFAT) that is known as the main regulatory enzyme of hexosamine pathway (4).

The aim of this study was to investigate the effect of vitamin D consumption on the serum levels of glucose, insulin, HbA1c, and AGE, as well as the expression of five previously mentioned key genes involved in the development of diabetes complications in liver tissue.

## Materials and Methods

***Animals***

Initially, 30 Sprague-Dawley male rats aged 3-4 months with an average weight of 300±40 g were purchased and kept in standard laboratory conditions at a temperature of 20-25 ^°^C and with 12-hr light/dark cycle for 10 days prior to the start of the study. Throughout the study, animals had free access to drinking water and standard laboratory chow containing 0.95 to 1.0% calcium and 0.65 to 0.70% phosphorus. Animals were cared in accordance with Guide on the Care and Use of Experimental Animals, and outmost attempt was made to minimize the number of laboratory animals and also to alleviate the pain and damage caused by the experiment (13). The experimental protocol was approved by the Ethical Committee of the Tehran University of Medical Sciences, Tehran, Iran. 

***Study design***

The rats were randomly divided into three groups and two rats were placed in each cage. At the beginning of the study, the rats in groups 2 and 3 received an intraperitoneal (IP) injection of Streptozotocin (STZ) (45 mg/kg, ~ 20 μl) and the rats in the control group were injected with the same volume of citrate buffer as placebo. STZ was purchased from Sigma (Sigma–Aldrich, St. Louis, Missouri, USA) and was dissolved in sterile sodium citrate buffer with pH of 5-6 immediately before injection. One week later, fasting plasma glucose (FPG) of all animals was measured by glucometer (Accu-check, Roche Diagnostic GmbH, Mannheim, Germany) and values greater than 250 mg/dl were considered as an indicator of diabetes. Finally, 8 rats that met the inclusion criteria were assigned to each group. Animals in group 3 on the 1^st^ and 14^th^ days of diabetes development received intramuscular (IM) injection of 20000 IU/kg vitamin D. Given that sesame oil was used for diluting vitamin D, it was injected to other groups as placebo. Food intake was checked and weighted per cage on a daily basis. The animals’ weight was recorded weekly and on the final day. After four weeks of vitamin D injection, all animals were anesthetized and sacrificed with IP injection of ketamine (50 mg/kg) and xylazine (30 mg/kg). Blood and liver samples of animals were immediately collected and kept in proper temperature.

***Biochemical tests***

Fasting blood samples were collected (between 8:00 to 10:00 AM) by cardiac puncture and centrifuged immediately to separate the serum. The serums and liver samples were immediately frozen in liquid nitrogen and stored at -80 ^°^C until biochemical assays. Serum levels of glucose, HbA1c and calcium were evaluated by auto-analyzer (Biotecnica Instruments, Rome, Italy). Vitamin D, insulin and AGEs concentrations were measured by commercial enzyme-linked immunosorbent assay (ELISA) kits (IBL international, Hamburg, Germany). 

***Evaluation of gene expression***

Initially, liver tissues were homogenized and mRNA was extracted using Hybrid-R RNA isolation kit (GeneAll Biotech., Seoul, South Korea). The amount and purity of RNA was evaluated by Nanodrop spectrophotometer (NanoDrop Technologies, Wilmington, Del., USA) and the ratios of 260/280 and 260/230 ~ 2.0 were considered as acceptable values. Then, cDNA was made and the expression levels of AGER, GLO-1, AR, OGT and GFAT genes were assessed by quantitative Real-time PCR using SYBR Premix Ex Taq II (Takara Bio Inc., Shiga, Japan). Sequences of primers were designed by using OligoCalc, Primer blast and GeneRunner softwares, and their specifications are presented in Table 1. Expression of mRNA was calculated with ΔCt method and glyceraldehyde 3-phosphate dehydrogenase (GAPDH) was considered as housekeeping gene.

***Statistical analysis***

Data were expressed as mean±SD and were analyzed using Statistical Package for the Social Sciences (SPSS, version 21.0; SPSS Inc., Chicago, Illinois, USA) and the graphs were designed by GraphPad Prism (Version 7.03; GraphPad Software, La Jolla California, USA). Kolmogorov-Smirnov test was used to assess the normality of the data. In order to remove the effects of confounding factors such as body weight and food intake, analysis of covariance (ANCOVA) followed by the Bonferroni *post hoc* test were used to compare statistical differences between the groups. Furthermore, the non-parametric data were transformed logarithmically for analysis. *P*-values less than 0.05 were considered as statistically significant.

## Results

At the beginning and end of this study, groups were compared in terms of body weight and food intake. No significant difference was found between groups at the beginning. However, the two diabetic groups had a lower food intake and body weight at the endpoint (*P* < 0.001; Table 2). Therefore, ANCOVA test was used to control the possible confounding effect of these factors. 

After four weeks of treatment, the mean FPG level was increased drastically in diabetic animals in comparison with the control group (*P*<0.001). Two injection of vitamin D resulted in a significant increase in plasma cholecalciferol level and could improve hyperglycemia and hypoinsulinemia in comparison with diabetic rats (*P*=0.005 and *P*=0.01, respectively). Calcium level was checked and hypercalcemia was not observed in any group. Serum HbA1c and liver AGE concentrations had a slight but insignificant decrease after vitamin D injection (Table 3).

**Figure 1:**
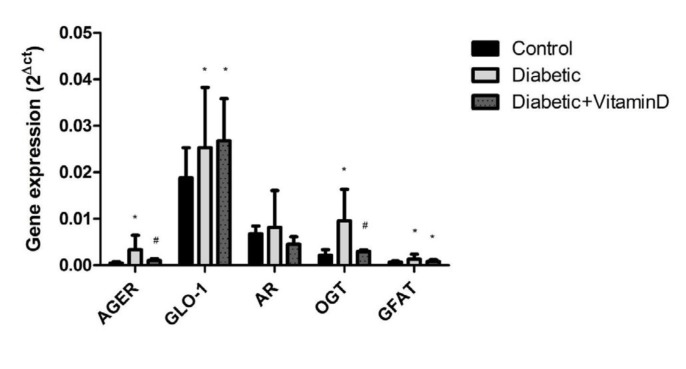
Gene expression of advanced glycation end products receptor (AGER), glyoxalase-1 (GLO-1), aldose reductase (AR), O-GlcNAc transferase (OGT) and glutamine: fructose-6-phosphate aminotransferase (GFAT) in the liver tissue of different experimental groups. Data are presented as the Mean±SD (n=8 for all groups). *, *P*<0.05 compared to the control group; #, *P*<0.05 compared to the diabetic group

**Table 1:** Primer sequences for real-time PCR

Gene	Sequence (5' → 3')	Length	Tm	GC%
AGER	F: ACAGAAACCGGTGATGAAGGA	21	59.3	47
	R: TCTCCTCGAGTCTGGGTTG	19	58.1	57
GLO-1	F: TGACGAGACGCAGAGTTACC	20	59.4	55
	R: GATCTTGAACGAACGCCAGAC	21	59.6	52
AR	F: AGTAGCTGAGGAGTTTCTTCG	21	57.1	47
	R: CATAGGACTGGAGTTCTAAGCA	22	56.9	45
OGT	F: AGCTCTTCGTCTGTGTCCTA	20	57.8	50
	R: CAAACTCTGGGAAGACCTCTA	21	56.3	47
GFAT	F: TTGATTCTGATTGCTTGTGGC	21	57.4	43
	R: ACAGTAGCGAAGACCCATCA	20	58.4	50
GAPDH	F: CATTCTTCCACCTTTGATGCTG	22	57.9	46
	R: TGGTCCAGGGTTTCTTACTCC	21	58.9	52

AGER, advanced glycation end products receptor; GLO-1, glyoxalase-1; AR, aldose reductase; OGT, O-GlcNAc transferase; GFAT, glutamine: fructose-6-phosphate aminotransferase; GAPDH, glyceraldehyde 3-phosphate dehydrogenase; Tm, primer melting temperature; GC%, primer guanine-cytosine content; F, forward; R, reverse

**Table 2:** Body weight and food intake of different groups

Groups	Initial weight (g)	Final weight (g)	Food intake (g/day)
Control	294.3±15.4	304.0±22.1	29.0±1.2
Diabetic	295.5±14.5	222.8±30.1*	22.9±2.4*
Diabetic + vitamin D	296.5±11.1	198.0±40.0*	24.9±4.0*

Data are presented as the Mean ± SD (*n *= 8 for all groups). Statistical differences were determined using paired t-test;

* , *P *< 0.05 compared to the control group;

# , *P *< 0.05 compared to the diabetic group.

**Table 3:** Biochemical factors in different experimental groups

Groups	FPG (mg/dl)	Insulin (mIU/l)	HbA1c (%)	Liver AGE (ng/ml)	Vitamin D (ng/ml)	Calcium (mg/dl)
Control	85.87±12.63	3.37±0.83	4.65±0.50	103.78±13.71	20.93±2.49	9.27±0.59
Diabetic	479.37±27.90*	2.15±0.79*	8.75±0.48*	123.98±10.79*	21.33±2.44	9.47±0.41
Diabetic + vitamin D	428.87±37.74*#	3.31±0.65#	8.30±0.55*	118.72±12.58*	35.42±3.96*#	9.52±0.95

Data are presented as the Mean ± SD (*n *= 8 for all groups). FPG, fasting plasma glucose; AGE, advanced glycation end products. Statistical differences were determined using ANCOVA followed by a Bonferroni *post hoc* test;

* , *P *< 0.05 compared to the control group;

# , *P *< 0.05 compared to the diabetic group.

Expression of AGER mRNA was dramatically increased in diabetic group (*P<*0.001). However, vitamin D could down-regulate it significantly (*P*=0.04). The expression of GLO-1, another key enzyme in AGE pathway, was elevated in all diabetic rats (*P*=0.02) and vitamin D treatment did not show any additional effect (*P*=0.7). Aldose reductase gene expression did not change neither in diabetic nor vitamin D-receiving rats (*P*=0.2). In the hexosamine pathway, OGT and GFAT expression was elevated in diabetic group (*P*<0.001 and* P*=0.02, respectively), but only OGT improved as a result of vitamin D injection (*P*<0.001; Figure 1). 

## Discussion

In recent years, numerous epidemiological and experimental studies have indicated that vitamin D deficiency can play a role in the incidence and progression of both type I and type II diabetes (14, 15). Although low levels of vitamin D have been observed in patients with diabetes, clear and sufficient data has not been obtained from clinical trials to confirm the causative or therapeutic effect of vitamin D in diabetes. According to the results of this study, two injections of vitamin D (20000 IU/kg) and its increased serum levels could lead to a significant reduction in blood glucose. This dose was chosen based on the results of our pilot study to elevate the serum level of vitamin D up to 30 ng/ml. In addition, HbA1c showed a moderate decline, which was not statistically significant probably due to the short period of study. It seems that substantial increase in the insulin level is one of the main reasons for the improvement of blood glucose in this experiment. Previous studies also suggest that adequate serum levels of vitamin D can protect pancreatic beta cells and help insulin synthesis and secretion (16-18). Also, other mechanisms have been proposed to explain the role of vitamin D in improving blood glucose levels. For example, it has been reported that vitamin D can increase the glucose transporter 4 (GLUT4) gene expression or improve the performance of glucagon-like peptide 1** (**GLP-1), and these pathways were not investigated in the present study (19, 20).

The results of this study also showed that vitamin D independent of its effects on the control of glucose metabolism might reduce the complications of diabetes by influencing cellular pathways like AGE. Previous studies have shown that there is a direct relationship between serum levels of AGE and the incidence of diabetic microvascular complications such as nephropathy, neuropathy and retinopathy (21-23). According to the results of this one-month examination, injections of vitamin D had no significant effect on AGE level in liver tissue. However, vitamin D decreased the expression of AGE cellular receptor gene. Therefore, it is expected that vitamin D may delay the complications of diabetes (24). The possible changes in chemokines, cytokines, adhesion molecules, and other compounds that increase following the activation of AGE pathway can be the subject of future studies. On the other hand, the hypothesis that vitamin D can impact on diabetes complications via the changes in the activity of glyoxalase enzyme (the enzyme that catalyzes and eliminates glycosylated compounds from the body) was rejected due to lack of change in GLO-1 gene expression. The level of this enzyme was elevated in all diabetic rats, which might be a protective response to hyperglycemia and activation of AGE pathway (25). Nevertheless, vitamin D treatment did not cause any additional effect.

Moreover, vitamin D had no effect on aldose reductase gene expression in liver tissue. Previous studies have shown that increased expression and function of this gene in diabetic people can increase the speed of incidence of diabetes complications, while inhibiting this enzyme can improve the patient’s condition (26, 27). Considering the fact that no significant change was observed in aldose reductase gene expression in diabetic rats, it seems that the duration of this study has not been enough to measure detectable changes in this gene. Besides, the possible protective effect of vitamin D was not also detectable.

Based on our findings, OGT enzyme levels had a significant increase in the liver of diabetic rats. This enzyme catalyzes the attachment of N-acetylglucosamine unit to the serine and threonine residues of proteins. Glycosylated proteins play a role in the development of diabetic complications (28). Moreover, it is likely that this enzyme by affecting the structure and function of proteins such as insulin receptor substrate 1 (IRS1) and serine/threonine-protein kinase 2 (AKT2) as well as by reducing insulin signaling cause insulin resistance (29). In addition, the results of Inbathamizh *et al.* analysis suggest that probably calcitriol in terms of three-dimensional structure and active sites might have an inhibitory effect on glucosamine transferase enzyme (30). In this study, vitamin D significantly reduced OGT gene expression in the liver. The expression of GFAT gene, as an important enzyme in hexosamine pathway, increased in diabetic rats and this change was not improved by vitamin D consumption within one-month study period. 

Examination of the weight and food intake of rats showed a mild decrease in food intake accompanied by a dramatic weight loss in diabetic groups. Hence, the role of these confounding variables was eliminated in all statistical comparisons.

The main strength of this study was to investigate the role of vitamin D in regulating gene expression of key receptors and enzymes involved in developing the long term complications of diabetes for the first time. However, measuring the levels of proteins and enzymes activity could certainly improve the reliability of present data and it may be the subject of future investigations.

## Conclusion

In summary, this study showed that injection of vitamin D and its increased serum levels lead to a significant improvement in blood glucose and insulin levels as well as a decrease in AGER and OGT gene expression in the liver of diabetic rats. Thus, it can be concluded that vitamin D may contribute in reducing diabetes complications mainly through AGE and hexosamine pathways.
